# The London Dissector, or Guide to Anatomy

**Published:** 1839-12

**Authors:** 


### THE LONDON DISSECTOR, OR GUIDE TO ANATOMY,

For the use of Students, &c., revised and corrected by EDWARD J. CHAISTY, M. D., demonstrator of Anatomy in the University of Maryland, 12 mo. p. p. 273. Baltimore, John Murphy, Printer and Publisher. 1839.

The character of the above work, is too well known to the medical public to need any recommendation from us. To the student of medicine, in the prosecution of his anatomical studies, it is of great value. In fact, it, or some similar work could not well be dispensed with ; and from the hasty perusal that we have given it, we hesitate not to say, that its value has been greatly enhanced by the revisions and corrections which it has received from Dr. Chaisty. The typographical execution of this work, is neat, as indeed is every thing, that issues from the press of Mr. Murphy.

